# Process evaluation for complex interventions in health services research: analysing context, text trajectories and disruptions

**DOI:** 10.1186/s12913-016-1651-8

**Published:** 2016-08-19

**Authors:** Jamie Murdoch

**Affiliations:** School of Health Sciences, Faculty of Medicine and Health Sciences, University of East Anglia, Norwich Research Park, Norwich, NR4 7TJ UK

**Keywords:** Process evaluation, Complex health interventions, Linguistic ethnography, Context, Randomised controlled trials

## Abstract

**Background:**

Process evaluations assess the implementation and sustainability of complex healthcare interventions within clinical trials, with well-established theoretical models available for evaluating intervention delivery within specific contexts. However, there is a need to translate conceptualisations of context into analytical tools which enable the dynamic relationship between context and intervention implementation to be captured and understood.

**Methods:**

In this paper I propose an alternative approach to the design, implementation and analysis of process evaluations for complex health interventions through a consideration of trial protocols as textual documents, distributed and enacted at multiple contextual levels. As an example, I conduct retrospective analysis of a sample of field notes and transcripts collected during the ESTEEM study - a cluster randomised controlled trial of primary care telephone triage. I draw on theoretical perspectives associated with Linguistic Ethnography to examine the delivery of ESTEEM through staff orientation to different texts. In doing so I consider what can be learned from examining the flow and enactment of protocols for notions of implementation and theoretical fidelity (i.e. intervention delivered as intended and whether congruent with the intervention theory).

**Results:**

Implementation of the triage intervention required staff to integrate essential elements of the protocol within everyday practice, seen through the adoption and use of different texts that were distributed across staff and within specific events. Staff were observed deploying texts in diverse ways (e.g. reinterpreting scripts, deviating from standard operating procedures, difficulty completing decision support software), providing numerous instances of disruption to maintaining intervention fidelity. Such observations exposed tensions between different contextual features in which the trial was implemented, offering theoretical explanations for the main trial findings.

**Conclusions:**

The value of following how trial protocols produce new texts is that we can observe the flow of ‘the intervention as intended’ across a series of events which are enacted to meet specific demands of intervention delivery. Such observations are not solely premised on identifying routines or practices of implementation, but where ‘protocols as intended’ breaks down. In doing so, I discuss whether it is here where we might expose the ‘active ingredients’ of interventions in action.

**Electronic supplementary material:**

The online version of this article (doi:10.1186/s12913-016-1651-8) contains supplementary material, which is available to authorized users.

## Background

The need for well-designed process evaluation of complex health interventions has arguably never been more recognised or of pressing importance. Ioannidis [1] discusses the waste involved in the 20,000 trials launched globally every year, citing evidence indicating that 34 % of completed surgical trials are not published. One possible reason for this finding could be that trialists did not wish to publish results that were non-significant or less favourable. While not publishing trial findings is unethical in itself [2], there is arguably an ethical obligation to embed a well-designed process evaluation within trials of complex interventions to enable researchers to provide explanations for the effects observed, offer suggestions for improving trial implementation, and to appropriately inform the implications for practice and future direction of research. The most recent publication of Medical Research Council (MRC) guidance on how to design process evaluations [3] marks a significant shift in this direction, recognising the importance of incorporating evaluative mixed methods into randomised controlled trials for the testing of complex interventions.

However, the challenge for such evaluations is how to integrate findings from data obtained using methods with very differing underpinning philosophies about the nature of the relationship between the observer and the observed, the role of context, the differing levels of analysis and ultimately the epistemologies that are produced as a result. Such tensions of data integration have already been debated and discussed at length with regard to mixed methods research broadly [4, 5]. The pragmatic challenge for process evaluation design is to reconcile these tensions so that plausible explanations for observed trial effects are provided and the contextual circumstances under which an intervention succeeds or fails are specified. This paper describes a conceptual framework for analysing the relationship between intervention and context of delivery at different levels of implementation, from a macro (infra-structural relations, broader cultural discourses, policies) level, which sets the broader context into which the intervention is to be tested, to its use at meso (institutional and interpersonal relationships) and micro (individual capacities) levels in its translation and implementation at the point of delivery.

### Defining context within process evaluations

There is a large body of literature that has theorised context [6] and particularly the relationship between language and context [7]. Definitions of context have varied enormously, from broad social forces [8] to cultural activities [9], to how particular words are uttered within specific interactions [10], presenting a difficulty for evaluation. MRC guidance on process evaluations states that context “may include anything external to the intervention which impedes or strengthens its effects.” [3]. Reports of process evaluations typically lack sufficient explication of what counts as context, how different contextual elements interact, and how to understand the relationship between context and the intervention itself [11].

However, theoretical perspectives are available for informing how to systematically investigate the implementation of interventions within context. In a review of research on the spread and sustainability of innovations within health service delivery, Greenhalgh et al. [12] identified how the relationship of innovations to context has evolved from reductionist views of individual responses to more complex considerations of wider political and ideological contexts, how the meaning of innovations as intended may differ from those adopting it within different contexts, and the importance of examining the interaction between innovations and the potential context. Such perspectives resonate with sociological models of innovations as ‘boundary objects’ [13], which consider processes of changing the meaning of objects across contexts to enable adoption by different groups. Similarly, Diffusion of Innovation [14], conceptualises how individuals, situated within a social structure, intersect with new ideas or practices through a sequence of events as they are implemented across boundaries of time and space.

There has been a growing recognition of the dynamic and evolving relationship between context and interventions [11, 15] within clinical trials. Critical realism [16] has increasingly been applied as a theory to underpin process evaluations, providing firmer foundations for the investigation of interventions within context. Critical realism recognises a social reality outside of individual perception, placing emphasis on how historical structures shape ongoing relations between individuals within institutional settings over time. Realist Evaluation [17], underpinned by critical realist assumptions, has recently been applied to assess the implementation of interventions within trials [18]. The approach is to establish ‘generative’ understanding of causality by identifying the mechanism that connects two events and the context in which that relationship occurs.

However, Realist Evaluation indicates a common feature of process evaluation design to date, in that contextual elements are presented within a relatively stable relationship between social structure and intervention delivery. In addition, process evaluations lack the tools to investigate context within a dynamic social structure. Instead they are designed to facilitate the use of methods that take snapshots of implementation. As a consequence, what we observe is what Fabian [19] referred to as a “timeless present” that “freezes a society at the time of observation.” Such limitations are reflected within conceptual frameworks such as the PARiHS [20] and the Consolidated Framework for Implementation Research [21], which despite conceptualising context in terms of a set of interacting constructs that influence implementation, do not provide the means to investigate this process. There is a need to translate conceptualisations of context into tools and analytical foci which enable the dynamic relationship between context and the implementation of interventions over time to be captured and understood.

### Using Linguistic Ethnography to examine the process of intervention delivery across macro, meso and micro levels of context

The emergence of Linguistic Ethnography (LE) in the United Kingdom [22] provides a particular operationalisation of critical realism [23]. LE has drawn on the respective strengths of linguistics to ‘tie ethnography down’ and ethnography to ‘open linguistics up’, offering tools for tracing a path between social reality and its enactment at a local level [22]. A key strength of LE is that it provides instructive theoretical and methodological concepts for the close investigation of context, which we can apply to clinical trial implementation. The work of Jan Blommaert [24, 25] has been key in these developments, particularly highlighting the limitations of taking discrete snapshots of events, and the importance of incorporating an historical perspective into our analysis of context. Blommaert pays attention to how the meaning of texts and behaviours shift as they interact with increasingly mobile and diverse environments, and emphasises how shifting discourse across time and space helps explain power inequalities in the production of meaning.

We can draw on Blommaert’s ideas when considering the relationship between complex interventions and context. One way to tease out this relationship is to begin with the premise that the intervention, prior to its implementation, manifests as a standardised document in the form of a trial protocol. To reach this point there will have been a long process of negotiation, debate, revision, re-negotiation from the initial conception of the idea, to the application for funding and ethical approval, through to the final protocol to be used within the trial. It has therefore been controlled and manipulated by scientists, funders, reviewers, policy makers and patients, and widely shared with those coming into contact with the study. The trial protocol is therefore a textual document that operates at a broad macro level across a range of settings that has emerged out of a specific historical context. The intervention is therefore a highly relevant element of context itself and cannot be seen as separate from the conditions in which it is implemented.

However, despite recent acknowledgement that protocols comprise both fixed and flexible elements [11], within the paradigm of positivist science and the epistemology of the randomised controlled trial, the trial protocol has to be viewed primarily as a non-contextual document, with transparent and stable meaning that, at its inception, is not intended to change. Within this view, an illuminating way to view the protocol is to draw on Blommaert’s notion of ‘fixed text’ [24] and to then examine what happens to the meaning of the text in its journey from inception to point of delivery. Protocols are translated/re-written in the form of standard operating procedures, personalised scripts and data collection sheets which are written with a view to performing particular activities. These texts are then enacted within those activities by a variety of people and likely to be interpreted and applied in diverse ways. The different texts produced therefore embody a history from the original fixed text of the trial protocol to the point of delivery itself, undergoing a process of selecting and designing meaning in texts that are intended to travel forward, but which also undergo a process of adaptation and re-interpretation by its recipients – a process of recontextualisation.

Analysing the process of how the fixed text of the trial protocol is translated into social action may enable researchers to assess how and whether the fixed immutable points of an intervention as intended are retained or transformed in the act of its delivery. One way in which this can be done is by examining how the language of the trial protocol is recontextualised firstly into language produced within other study documentation and then into talk and behaviour produced when those documents are used. This analytical approach has been referred to as ‘text trajectory’ analysis and has been applied outside of health research in studies of communication in a range of institutional settings [24–28]. Text trajectory analysis helps the researcher understand the flow of information and transformation of meaning and in particular, facilitates insight into events that have not been directly observed, in other words, a point B, between Point A and C, thereby offering a traceable device for the study of *process* without the necessary inferences required from taking a series of observations or interviews. Kell referred to these flows of events as “a set of emergent social processes unfolding over time and space, drawing on multi-modal forms of communication” [27].

To systematically examine the text trajectory of a trial protocol we can structure our analysis within a conceptual framework that sets out which elements of context operate at which contextual levels and then to analyse how the meaning of protocols, initiated at a macro level of context, translates across each contextual level. In a similar way to Realist Evaluation [17], Harris and Rampton [29] set out four elements of context which capture the dynamic and historical relationship between social structure and social action, which I have adapted here for this article’s focus on the implementation of complex health interventions:

1. *Macro:* Broader discourses, policies in play during trial implementation, infra-structural relations;
2. *Meso:* Institutional, network relations, histories of relationships and interaction prior to implementing the intervention;
3. *Micro non-linguistic:* Types of activity (i.e. tasks, ‘what are we doing here?’) participants are engaged in and interactional arrangements of intervention delivery; and
4. *Micro linguistic and non-linguistic:* Acts (specific actions within activities) and utterances involved in intervention delivery.

We can view each element of this contextual framework as interrelated in producing moments of intervention delivery. Participant (doctor, patient, nurse etc.) relations and histories of interaction will inform how the intervention is integrated into routine practice, how it is discussed and therefore the particular nuance given to the activity of intervention delivery. The activation of particular discourses when the intervention is implemented, meaning systemic, culturally-circulated explanations (for example, a broader discourse of what it means to provide continuous care to patients) will influence both how participants make sense of this kind of social activity and the ongoing sequences of interaction within it.

### Analytical focus of text trajectory analysis

The consequences of setting out a spatial-temporal framework of context and the implementation of health interventions as one in which language and social action are viewed as interconnected is that it suggests an alternative way to observe the implementation of interventions. Researchers typically obtain a series of discrete snapshots and then compare them looking for patterns and routines in how it is implemented. While this approach provides invaluable “thick descriptions” [30] it has the potential to gloss over how events come about, how they are historically constituted. One way of empirically observing the connection between macro, meso and micro is not only by examining how interventions are routinised in practice, but also in observing where implementation and integration is disrupted or breaks down. This is crucial as it exposes the wider social forces structuring intervention delivery *at the point of delivery*, relations which are otherwise hidden from view, as Wittgenstein discusses:

“The aspects of things that are most important for us are hidden because of their simplicity and familiarity. (One is unable to notice something - because it is always before one’s eyes.) … And this means: we fail to be struck by what, once seen, is most striking and most powerful.” [31]

Viewed in this way social structure is not just an organising feature of context, but is produced and observable *through* moments of intervention delivery. The moment of disruption is therefore a product and an opening to view social structure that would otherwise remain hidden. This exposes the program theory itself to observation, defined here as the explanation for why the observed effects of the interventions have been observed [32], and the circumstances under which the intervention may or may not be successful. We therefore have the potential to offer theoretical generalisibility, in that we can infer that if the intervention is similarly structured within the different sites of delivery then we are likely to observe similar forms of social action. In addition, it offers the opportunity to see how the theory might be improved by showing how adjusting a particular feature of context might lead to a different intersection of individuals, social structure and activity.

However, another important distinction between previous process evaluation design and the approach set out here is that by deploying a ‘text trajectory’ analysis of a trial protocol we are making the history of the intervention a unit of analysis. This goes beyond a chronological description by revealing how the creation of texts, (such as standard operating procedures designed to carry out a particular activity), provide the pre-conditions for potential disruptions to occur during implementation. The intervention being delivered at any point in time flows from a series of events that led up to that point, and which also involved the distribution and transformation of the protocol into different texts. These texts, when brought into use (or not) by participants, are individual events considered to represent the original protocol in some way. Yet the intervention that is actually delivered is a result of the whole trajectory of this process. There is therefore potential value in paying closer attention to the creation and enactment of texts and in focusing on disruptions, we are looking for clues of how the different ingredients of an intervention are functioning together. I have already highlighted the value of this approach in how people talk about medicine-taking [33, 34]. However, to demonstrate an example of the text trajectory of a trial protocol and how an analysis of disruptions exposes the ingredients of interventions, I will draw on data from a large cluster randomized controlled trial of telephone triage in primary care.

## Method

The ESTEEM study [35, 36] was a cluster randomised controlled trial which compared effects on primary care workload, cost, patient experience of care, patient safety and health status of computer-supported nurse-led telephone triage, GP-led telephone triage, and usual care. While the program theory for ESTEEM was not explicit, the premise of the trial rested on a rationale that assessment of urgency via GP and/or nurse-led telephone triage could reduce primary care contacts over 28 days. However, the ESTEEM findings revealed that, while nurse triage could offer a useful approach to support GP availability, both nurse triage and GP triage led to an increased rate of primary care contacts over 28 days, compared with usual care, including the initial triage contact [35]. In addition, it was found that only 12 % of patients in the nurse-triage arm had just one contact.

ESTEEM recruited 21,000 patients requesting same-day appointments in 42 General Practices across four different regions of England. Practices randomised to one of the two triage arms typically ran the intervention for a period of 2–3 months. The GP and nurse triage interventions were complex interventions that involved staff training (clinical and technology based); a computer decision support software (CDSS) to support the delivery of nurse triage; process and organisational change in practices regarding reception activity and appointment system management; and accommodation of patient expectations. Some core elements of triage delivery were common to, and adopted by, all practices in both intervention arms. However, some organisational flexibility was permitted so that local practices could manage the trial within the constraints of their day-to-day work.

All patients contacting the practice initially spoke to a receptionist. Once the receptionist established that the patient (or a proxy asking on their behalf) was requesting a same-day, face-to-face appointment with a GP, the patient was asked to provide a contact telephone number and was advised that the clinician (GP or nurse, according to the practice’s allocation) would call them back within around 1–2 h. This timescale was suggested as a guide for practices but was not considered mandatory.

A parallel process evaluation was undertaken [36, 37] which included 49 h of non-participant observation and 99 interviews with staff [see Additional file 1] and patients [see Additional file 2] in eight general practices. The process evaluation consisted of two discrete phases; an initial 1-year pilot study conducted alongside, and feeding into the pilot of the main trial, followed by the main process evaluation study. For the purposes of this paper, I draw on field notes of observations conducted during the set up and pilot phase of the trial in one nurse-led triage practice. The aim of the process evaluation pilot was to (1) ensure that the triage training and interventions could be feasibly delivered by practices and (2) test and fine-tune data collection methods. Observations were one week long and conducted by a trial researcher. In addition, a qualitative sub-study [38, 39] was carried out of 51 audio recordings of GP and nurse telephone triage consultations in two practices, including 10 video recordings of nurses’ use of CDSS during triage calls. For the purposes of this paper I draw on a multi-modal transcript of one nurse-led triage call, obtained from a separate practice to the field note data. In addition, I have retrospectively mapped out features of context for the ESTEEM trial at macro, meso and micro levels. In practice however, this work would be carried out prospectively, enabling the researcher to pinpoint key points of set up and delivery which warrant in-depth investigation.

## Results

In Tables 1, 2 and 3 I have set out the first three elements of context before presenting the extract [see Table 4] from the field notes and then the multi-modal transcript of a nurse-triage interaction [see Additional file 3: Table S1]. The field notes and transcript are from two different practices delivering nurse-led triage but provide examples of how the contextual elements played out at different stages of the ESTEEM trial. In doing so, we can consider the processes of recontextualisation of the protocol text and the implications this has for the delivery of the intervention.

**Table 1:** Macro level of context: discourses, policies in play during trial implementation

Type of macro discourse, policy in play	Description
Trial protocol as discourse	Telephone triage as research priority. Standardised ‘fixed text’ stating requirements of trial participation, integrity to intervention/control arms and fidelity, inclusion/exclusion criteria, ethical procedures and data collection.
Wider cultural discourse of telephone triage being about managing demand for care	Implementation of telephone triage in each GP practice is set with an agenda to reduce workload on healthcare resources. Triage interactions are therefore set up to assess urgency.
Telephone triage involves risk-minimisation	Introduction of telephone triage to manage acute cases necessitates a risk-minimisation approach. CDSS designed with clinical algorithms to minimise risk of clinician reaching incorrect triage outcome.
Nurse status/role and responsibilities	Historical discourse of primary care nurses building ongoing empathic relationships with patients, responsible for face-to-face chronic illness reviews. Telephone triage reconfigures role to gatekeeper/assessor of urgency, communicating with patients remotely to manage acute cases.
Patient-centred discourse of consultations	Consultations should be orientated towards patient-agendas and patient needs. Practitioner-patient relationship key to meeting patient’s needs.

**Table 2:** Meso level of context: Institutional, network relations, relationships and interaction history prior to implementing intervention

Institutional relations, histories, local policy	Description
History of interactions prior to implementation	Practice staff trained on study protocol and procedures as well as specific guidance on how to manage telephone triage appointments during trial; Receptionists received specific guidance with script on how to introduce triage to patients; nurses received training to use computer-decision support software; Research team liaised with practice staff throughout set up and delivery of trial. Individualised procedures were provided to all staff.
Institutional and network relations	Prior to ESTEEM, patients telephoned or visited surgery to book a same-day GP appointment. Following introduction of intervention, patients’ expectations of accessing care briefly re-oriented to telephone triage by receptionist.
Local policy on patient management	Practice specific procedures on managing telephone triage – e.g. triage sessions, staff allocation – nurse practitioners, practice nurses

**Table 3:** Micro level of context: Activity types participants engaged in and interactional arrangements of intervention delivery

Activities, interactions involved in intervention delivery	Description
Main activity	Delivering telephone triage to patients requesting a same-day appointment with a GP. Patient calls the practice requesting SD appointment, receptionist puts them on a list for nurse/GP to call back; nurse/GP calls back to triage patient. If nurse, then uses CDSS to triage patient. Patient either booked into SD appointment with GP/nurse, given appointment on another day, given self-care advice, or other outcome.
Subsidiary activities	Receptionists follow script to speak to patients and determine eligibility, then if eligible, flag up on appointment screen for triaging clinician. Receptionists complete log sheets during audit and run-in phase. Clinicians to complete clinician form to record details of call. Practice Manager to collate numbers of eligible patients and numbers receiving intervention.
Interactional arrangements	Telephone triage comprising one-to-one interactions utilising focused questions directed at caller about patient’s presenting problem. In nurse triage, interactions are guided by CDSS.
Interactional expectations/understandings	Understandings of purpose of telephone triage may be diverse across patients, thereby influencing their expectations and how they participate in triage interactions.

**Table 4:** Extract from field notes: processes of recontextualisation in the trajectory of ESTEEM trial protocol in one nurse-led triage practice

Stage in research process	Task	Texts used	Researcher’s fieldnotes - verbatim	Moments of vulnerability to processes of recontextualisation
Post-randomisation set up and training phase	External consultant training of GP practice on how to organise triage appointments.	Spreadsheet showing audit of practice’s same-day (S-D) appointment requests against available clinicians, and resources required to deliver triage. Flowchart of how S-D patients should be managed by practice. Ensure fidelity to eligibility criteria	Trainer shows graphs etc. on laptop computer; best suited to smaller groups. Solutions to potential logistical,/psychological barriers are proposed/discussed. Trainer shows a sample receptionist flowchart, suggesting practices devise their own. Practice manager and senior receptionist attended. Manager reported how triage patterns could be fitted into current practice consultation pattern.	Staff responses to audit critical in determining resource allocation to support triage. How receptionists respond to flowchart as text determines adherence to inclusion/exclusion criteria.
Post-randomisation set up and training phase	Training of reception team on research procedures by ESTEEM team members	Log sheets for recording how S-D appointment requests are managed by reception; personalized procedures for each receptionist detailing intervention process, including script when speaking to patients	Trial Manager reiterated triage numbers/processes, outlining log sheet completion, arranging log sheet faxing and READ coding of triaged patients’ notes. Practice manager and senior receptionist attended. The practice manager seemed engaged/enthusiastic, the senior receptionist less so. The trial manager explicitly reiterated the importance of triaging children. The practice manager agreed [in the meeting] to do this (but to make children covert appointments, for cancellation if not needed).	Log sheets emergent as text to monitor, regulate and standardize inclusion and exclusion of patient requests. Non-completion means fidelity can’t be assessed. Process of READ coding, faxing represents production of texts to show captured sample. Vulnerable to inaccuracies, missing data.
Post-randomisation set up and training phase	Training of nurses on research procedures by ESTEEM team members	Personalized research procedures for each nurse, emergent as text to ensure fidelity to intervention delivery. Case report ‘clinician’ forms, a key text in capturing trial outcomes	Three nurses attended, 2–3 will triage. Trainer talked through triage process and completing clinician forms. Nurses understood, but [as reported] confidence with triage varied.	Ability to follow procedures and accurately complete forms subject to time constraints.
Post-randomisation set up and training phase	Nurse training on use of CDSS, delivered by organisation providing CDSS for trial	CDSS as text, emergent out of trial philosophy of triage as means of managing demand.	Observed one online 1:1 interactive software training session for 2 h with one nurse. The nurse struggled, due to limited computer skills and frequent software crashes.	Nurses’ IT skills and confidence, functionality of CDSS key influences in how CDSS is used and triage delivered.
Run-in period: four week period where practices rehearsed delivering triage and completing research procedures before live data collection.	Receptionists’ identification of eligible triage patients	Personalised script produced by practice staff	During the run-in (observed over three separate days) staff used a practice-generated script which included the terms ‘triage/clinical assessment’. Explaining these terms led them to field patients’ questions/concerns, causing stress and reducing call handling rates. Without ESTEEM materials to hand, receptionists were panicked about who to triage. Feedback of this observation led to the provision of a new version of the triage criteria. During data collection the new ESTEEM triage criteria were to hand. Receptionists were calmer, although still not totally sure about who to triage.	Practice recontextualisation of script consequential for how patients receive triage and for further procedural iterations. Availability of research procedures key in how receptionists screened patients for eligibility.
‘Live’ implementation of nurse-led triage	Receptionists’ use of data collection log sheets	Log sheets for recording how S-D appointments are managed by reception team	Receptionists were not using the provided log sheets, and had not done so since ‘going live’. The practice manager found sheets and gave them to receptionists to use. Receptionists understood how to use the sheets but did so with varied completeness under pressure on the phone to patients. The practice is triaging only the first 10 eligible patients a day, due to limited triage nurse time (3 h are set aside daily). After all nurses’ triage slots were taken, receptionists were unsure whether to fill in all log sheet columns for eligible patients. Receptionists were unsure whether to give patients approximate times for nurse call back. Triaging children: The practice is not currently triaging children, despite explicitly agreeing to do so. Potential patient avoidance: One patient asked if she could avoid the new triage system by booking in person at the reception desk. The receptionist said yes.	Log sheets absent from delivery and fidelity to inclusion/exclusion criteria unclear. Process of ensuring protocol fidelity through training and provision of procedural documents failing to be enacted in delivery.

How the different elements of context within Tables 1, 2 and 3 circulate across practices and between practice staff and patients are key to understanding how the intervention was enacted, with numerous opportunities for the ‘meaning as intended’ being unevenly distributed across those who need to use the intervention. Using this contextual framework we can now examine the field notes [see Table 4] from the observation of the different stages of set up and delivery of the intervention in one nurse-led triage practice. At the time the observations took place, the analytical focus was to use a thematic analytical approach to identify factors influencing implementation, rather than how the trial protocol was being recontextualised in practice. The extract presented is not intended to represent how triage was responded to widely, however, the field notes provide some insight into this process of translation from standardised protocol as fixed text, to its use at both the meso and micro levels of context, and indicate moments where the delivery of the triage intervention were vulnerable to processes of recontextualisation which may have disrupted its implementation as set out within the trial protocol.

We can see from the brief notes within the field notes extract [Table 4] numerous indications of where the translation of the trial protocol as fixed text is vulnerable to processes of recontextualisation, in terms of: the interactional work in ensuring protocol is enacted by practices; skills required to deliver the intervention; ability to accommodate change into current practice appointment systems; participant’s recontextualisation of study terms and documents; technology; and deviations/breaches from the protocol. These vulnerabilities in translating the protocol as macro discourse to a meso level, are exposed as disruptions to intervention delivery exposing particular tensions between different social forces. In the extract this can be seen by how the protocol conflicts with a macro patient-centred discourse on the management of children, enacted at a local level (agreement to triage children with condition of covert appointments, but not enacted in practice); and through tensions between previous and current arrangements for booking an appointment (receptionist allows patient to bypass triage system).

These examples resonate with Normalisation Process Theory’s notion of embedding and integration of interventions into routine practice [40]. However, viewed as a fixed text on a trajectory, we can observe how the protocol is enacted at different points of delivery; how competing demands of the immediate interactional context (e.g. calls waiting, other patients at receptionist desk) may override the demands of the protocol; and the power (or lack of) different texts have in shaping staff actions, which in this example could be seen to be noticeable by its absence (receptionists panicked without ESTEEM guidance) while at other times ignored (completion of log sheets).

How the intervention is embedded and integrated is shown in this example to be fragmented by how staff deployed different textual documents. In making these observations we are able to assess whether fidelity to implementation of the intervention was maintained in this specific GP practice. However, we are also able to view these disrupted activities as revealing tensions between the different contextual features that were mapped out in Tables 1, 2 and 3. The advantage of prospectively mapping context then is to pre-empt where such tensions might occur, and then using text trajectory as a device, allow close investigation of whether and how such tensions are consequential for intervention fidelity.

However, disruptions to activity need not only be viewed in terms of evaluating intervention fidelity. By exposing how different contextual features intersect with one another we are exposing a particular relationship that allows us to re-evaluate our program theory. To obtain a more nuanced insight into the journey of trial protocols and what this might add to our understanding of theoretical fidelity [41] we can also analyse how texts are actually used as part of specific interactions and the consequences of their use for intervention delivery. While such data were not obtained within ESTEEM prior to implementation, recordings of nurse triage interactions were obtained in addition to video screenshots of nurses using computer decision support software. A supplementary transcript is provided [see Additional file 3: Table S1] as an example.

The purpose of analysing nurse triage recordings was to assess how the institutional requirement to manage patients, using CDSS, structured the triage calls and how this structure was consequential for how interactions proceeded and information obtained from patients. The CDSS therefore represented another text through which the triage intervention was mediated and delivered. Through an analysis of both interactional patterns and disruptions, we identified evidence of the CDSS as text playing a fundamental role in organising nurse’s questioning which at times adversely affected the information collected from patients [38, 39].

In the transcript [Additional file 3: Table S1] we can observe interactional difficulties throughout the sequence as the nurse can be seen to be negotiating what the patient is saying with what the CDSS is prompting as questions and offering as responses. These difficulties are not merely instances of trouble of using the technology but provide instances of disruptions to the intervention delivery which expose tensions between wider macro discourses that are set out in [Table 1] as structuring triage interactions. Firstly, the patient describes a history of back pain symptoms that have moved across her back in the last few weeks. However, the CDSS initially requires a response that focuses on one symptom, in one area and specific for that day only, reducing the patient’s presentation to pain in her lower back. This manifests in the interaction at 00:36 to 00:56 s and at 00:49 s we can see the nurse attempts to orient the patient towards this agenda ‘So it’s what you’re phoning up today with…’ . The patient then makes a request for painkillers, having earlier offered a candidate diagnosis of sciatica. However, the nurse’s statement that a review is required has the effect of dismissing this request, switching the genre of the interaction to hers and the CDSS agenda of history-taking questions.

Once the CDSS directed activity of history-taking is initiated, the nurse asks a question at 01:02 about the location of the pain, linked to pop-up box options in the CDSS which do not include an option for buttocks. Subsequently, when the patient says buttocks at 01:16, the nurse clicks ‘lower back’. At 01:21 the nurse offers the patient a CDSS prompted choice about how the pain started – “was a gradual on↑set or did it just suddenly sta::rt.” Following the patient reporting the pain suddenly started the nurse clicks ‘instant’ on the CDSS. However, this triggers a red category marker (the word ‘instant’ is highlighted red and a red square next to text) which means the patient needs to be seen now or within 1 h. This leads the nurse to change the response to ‘gradual’, which downgrades the urgency of the problem. Once back pain has been established as the key symptom, the CDSS prompts a question at 01:36 about the severity of the pain, requiring the patient to score their pain between 0 and 10. However, the patient’s response to the nurse’s phrasing of this question ‘my pai::::n?’ indicates she does not understand the nurse’s request for a number between 1 and 10. In response to the patient saying ‘it’s still there’ the nurse records ‘5–6: Moderate’.

Finally, at 01:54 the CDSS is prompting the nurse to ask about radiation of pain and the pop-up box includes ‘buttocks’. However, despite the patient earlier reporting pain in the buttocks as a key symptom, the nurse’s question leans towards a negative answer, which the patient appears to have difficulty responding to with the response of the presence rather than absence of pain – “We::ll [I’d say it] (0.6) well I don’t kno::::w it =” At this point the nurse initially records the answer as ‘unsure’ but then switches the response to buttocks.

Evident within the different instances of interactional trouble we observed were three distinct speakers (nurse, patient, CDSS) which could be seen to move in different directions, exposing wider social forces which pass by imperceptibly when such interactions run smoothly. These are that triage is about efficiently determining whether the patient should receive a face-to-face appointment; that consultations should be patient-centred and patient’s needs must be met; and that the institutional requirement for nurses to triage patients on the telephone, using CDSS, exposes the governing and standardising effect of the CDSS for how patients come to be categorised as levels of ‘risk,’ underpinned by concerns of litigation and accountability.

These instances of interactional trouble, viewed as disruptions at a micro level, exposed tensions and contradictions in these social forces and, by doing so, provide the basis for one theoretical explanation for why only 12 % of patients within the nurse triage arm had only one contact. This is that when delivering triage, using CDSS, within any GP practice, nurses will have had to negotiate tensions between different social forces of risk minimisation, patient demand, accountability and patient-centred needs. We can therefore make a theoretical generalisation that, as a consequence of needing to manage these tensions, nurses struggled to perform the twin parallel tasks of attending to patient’s needs and accurately completing the CDSS. A hypothetical consequence of these interactional dilemmas was that nurses frequently opted for the safest option, which was to book patients a face-to-face appointment with a GP.

This theoretical explanation is likely to be only one part of a broader explanation of why so few patients in ESTEEM had only one contact. However, the insights provided within this analysis were not provided by obtaining detailed descriptions of typical cases. Rather it was through a consideration of how the decision to employ a CDSS to mediate the nurse-triage intervention introduced unforeseen tensions to delivering the intervention when later enacted by nurses. Such tensions were then revealed through identifying ‘telling cases’ [42], where a type of ‘disruption’ or interactional trouble occurred. By prospectively mapping the CDSS as a text, which embodies particular contextual features through its design, we might have identified how its use may have compromised the fixed and immutable elements of the triage intervention.

## Discussion

Setting out the contextual conditions under which a trial is implemented, at a macro, meso and micro level, provides a framework for tracing the trajectory of trial protocols, designed as ‘fixed texts’, through to the point of delivery. The protocol, developed through a process of debate, negotiation, revision and critical review can therefore be viewed as emerging out of a particular social historical context and implemented across a range of settings and with patients with diverse backgrounds. Viewed at a macro contextual level we can then see the standardised elements of protocols distributed across a range of texts at meso and micro levels – audit and log sheets, research procedures, data sheets and mediated via technology, all which may be responded to by participants in a variety of different ways. It is the power afforded to such texts that is pertinent in this regard, as they are the means by which the practices and procedures required to deliver a trial are intended to be sustained.

Analysing interactional data for ‘disruptions’ in activity at a micro level, enables tensions and contradictions in this process of recontextualisation to be exposed. Importantly however, these tensions and contradictions are historically constituted, tracing how shifts in discourse, network relations and activity interact with the histories of staff and patients who need to move along with these changes. Viewed in this way, the delivery of an intervention cannot be seen as a discrete object that is standardised and implemented across a range of settings. Instead, it can be seen as made up of multiple layers of discourse circulating along different historical processes, that come together at any particular instant, what Blommaert described as ‘*collapsing in synchronic moments of occurrence*’ [25]. Analysing the trajectory of texts enables us to examine how such historical processes are enacted within such moments of occurrence. In the case of nurse triage, observing interactional disruptions exposed the organising effect of the telephone triage intervention, recontextualised from standardised protocol at a macro level, to its delivery at a meso and micro level, revealing the dynamic and evolving nature of the triage intervention across different contextual levels. At each point in which we observed the delivery of triage, we were not observing the original protocol, but an intervention as a ‘critical event in the history of a system’ [43].

While we were not able to speak for how all such triage consultations were conducted using CDSS, either in this particular GP practice or beyond, we were able to argue that we observed a particular structural relationship that is likely to be replicated in other GP practices where nurses are required to use the same CDSS to triage a diverse range of patients over the phone. Such insights can then offer theoretical explanations that such contextual conditions may produce particular outcomes, in this case potentially explaining the high proportion of nurse triage patients converted to face-to-face appointments. By implication, we might then also speculate that by adjusting any one of these contextual elements at either macro, meso or micro levels, we might then observe different outcomes for patients. For example, removing the requirement for nurses to use CDSS would have withdrawn CDSS-directed questions orientated towards a risk minimization discourse. While triaging patients over the phone may have meant nurses would continue to orientate to risk-minimisation, this different interactional space may have facilitated a more patient-centred discourse to circulate between nurse and patient, potentially influencing how patients were triaged. Indeed we have already observed such interactional differences between nurses and GPs when GPs were not using CDSS to triage patients [39].

Engestrom [44] argues that only by mapping out historical changes in the activity system of doctors against substantive dimensions of the medical encounter can we move beyond ahistorical analyses, such as seen in Conversation Analysis [45] which typically reproduce findings on asymmetry between doctor and patient. By setting complex health interventions and specifically trial protocols within a historical context we are able to make the transition from how interventions are locally integrated and embedded within particular sites to an explanation of structural relations beyond the specific setting. Instead of offering an explanation of how interventions operate as comprising a set of contextual features signifying success or failure, our explanation is a discussion of how moments of intervention delivery are inextricably linked to wider social forces that will also organise how the intervention is delivered in other contexts, thus enabling a different form of generalisation beyond that immediate context. In critical realist terminology, such generalities have been coined ‘demi-regularities’ that indicate ‘the occasional, but less than universal, actualization of a mechanism or tendency, over a definite region of time-space’ [46]. At the point of intervention delivery, the analyst is not just looking at an individual act that is discrete and separate from other moments of social action. Rather, the language or act is a product of and productive of wider historical discourses and structures in which the delivery of the intervention can be seen. This is not the same as saying that all individuals or settings are the same, and I have argued how context needs to be investigated at multiple levels. Rather it is that social action in one circumstance is connected with social action produced in another through social contexts that are shared and that can be seen to be manifested in the act of delivering the intervention. Setting out the contextual elements at different levels points the way for examining this connectivity and where we might else look for data that might also expose how other ingredients interact.

Viewing the delivery of interventions in terms of historically constituted activities with internal tensions and contradictions therefore contributes to our understanding of whether and how we can observe the active ingredients of interventions. Wells et al. [11] argue that:

“complex interventions in healthcare are built up from a number of components, which may act independently or interdependently although the ‘active ingredient’ is generally difficult to specify. The components usually include behaviours, characteristics of behaviours (for example, frequency, timing), and methods of organising and delivering those behaviours for example type(s) of practitioners, setting and location.”

Viewed as a product and productive of a particular social historical context, the active ingredients can be seen as particular structural relations that organise intervention delivery in particular ways, but which are still subject to how they intersect with the histories of those delivering and receiving it. The ingredients are only active if the intersection of discourse, activities and participant histories function to produce an outcome as intended by those designing the intervention. It is therefore perhaps a mistake to think of interventions as having decontextualized ‘active ingredients’, but instead can only be viewed as contextually and interactionally produced ingredients which organise intervention delivery produced in the process of implementation.

However, the approach set out in this paper is not intended to replace a more conventional approach of mapping a field of study and identifying patterns of use. Conducting ethnographic fieldwork to obtain a ‘thick description’ [30] of intervention delivery, or eliciting individual’s perspectives undoubtedly offers insight into the routine use of an intervention, and helps identify the contextual conditions of success or failure of intervention. In looking for where the delivery of an intervention is disrupted in some way, the analyst inevitably runs the risk of missing a particular configuration of contextual features which may enhance intervention fidelity. Nevertheless, using text trajectory analysis as a device for identifying disruptions goes beyond identifying what works for whom and in what circumstances. Evaluating the production of texts, and how they are enacted, exposes contextual forces to observation, allowing us to scrutinise our program theory of the intervention. We can answer questions about *how and why* we can observe an intervention working or not working as inevitably produced by, and through particular contextual conditions. Acknowledging this view ultimately offers a challenge to trial design itself, posing whether the effectiveness of complex interventions can only ever be understood as activity produced through context.

## Conclusion

For those designing process evaluations, a key issue in considering the approach set out in this paper is how does an examination of the trajectory of protocols, viewed through instances of disruptions to activities, contribute something different to other approaches and what are the costs and benefits of conducting this type of analytical work? To observe the function of texts within interactions, not only in the delivery of interventions but in the set up and training phases, requires a level of involvement in trial implementation that goes beyond ethnographic methods that have been employed for process evaluations [47]. However, the strength of examining the trajectory of trial protocols is that exposing the social forces organising intervention delivery requires an analysis of ‘telling cases’ [42] rather than ‘typical cases’. Instances of interactional disruption offer such cases and therefore do not necessarily require a large sample in which to observe them or a greater burden on research resources to conduct such an analysis. Instead of aiming to identify routines, patterns and processes across a range of cases, a text trajectory approach would require an examination of the social structuring of interactions. This strategy has already been argued within critical realist evaluations [48] and proposals to adopt to ecological approaches to evaluation [49] which considers the implementation of interventions from a systems perspective. What is offered in this paper builds on these perspectives by offering a means of prospectively planning and systematically identifying how relationships, roles and moments of delivery are organised by, and expose social structure. It offers a strategy for operationalising an evaluation of implementation fidelity, but also theoretical fidelity. This is proposed by: 1) setting out macro, meso and micro contextual features before trial implementation; 2) targeting where likely tensions are likely to occur between different contextual features, including a consideration of the trial protocol as a contextual feature itself; then 3) searching for disruptions in targeted activities; and 4) considering the consequences of these disruptions for how the trial was conducted and the implications of these consequences for observed effects of the main trial findings.

## Additional files

Additional file 1: Staff interview guide. Description of data: Topic guide for interviews with staff participating in ESTEEM process evaluation (DOC 31 kb) Additional file 2: Patient interview guide. Description of data: Topic guide for interviews with patients participating in ESTEEM process evaluation (DOC 295 kb) Additional file 3: Table S1. Analysing disruptions in the implementation of primary care telephone triage. Description of data: Multi-modal transcript, including use of screenshot images (DOC 290 kb)

## Abbreviations

CDSS: Computer Decision Support Software
ESTEEM study: Name for a Cluster Randomised Controlled Trial of Primary Care Telephone Triage
GP: General Practitioner
LE: Linguistic Ethnography
MRC: Medical Research Council
