# Supplementary material for: Process evaluation for complex interventions in health services research: analysing context, text trajectories and disruptions
Source: BMC Health Serv Res. 2016 Aug 19;16:407. doi: 10.1186/s12913-016-1651-8 (PMC4990981; doi:10.1186/s12913-016-1651-8)
Supplement: Additional file 1: — Staff interview guide. Description of data: Topic guide for interviews with staff participating in ESTEEM process evaluation (DOC 31 kb) [file 12913_2016_1651_MOESM1_ESM.doc]

**Staff Interview Guide: Intervention Group**

## Preamble:

- Thank staff member for agreeing to meet/their time etc.
- Outline the pilot study aims.
- Check staff have read/understood the study information sheet.
- Give them a chance to ask questions.
- Check that they wish to proceed with the interview.
- Complete/sign two consent forms (leave one with staff).
- Advise staff that we seek their experience/views, with no ‘right’ or ‘wrong’ answers.
- Advise them they can stop the interview at any point/decline to answer any questions.
- Advise them when audio-taping will begin.

## Interview questions:

The ESTEEM trial looks at what happens when patients ring the surgery for

‘same day’ consultations using the new telephone triage system.

1. Can you tell me in what ways are you involved with the new triage system?

- prompt for role within the practice

2. Can you describe how the new system has been set up in your practice?

- prompt for detail

3. Can you describe how the triage system operates here?

4. Have there been there any problems setting up the triage system?

5. Have you managed to solve these problems? If so, how … ?

6. How are you finding the new triage system generally?

7. How well do you think the new system works for patients?

8. How well does the new system work for you?

prompt – any experienced/perceived advantages/disadvantages triage?

9. How well do you think the new system works for other staff?

10. Are there any particular strengths of the new system?

11. What do you think are the weaknesses of the system?

12. Would you be willing to keep on using the new system once the study is over?

13. Did you get any training in using the new triage system?

14. How well do you feel the training prepared you to run the new system?

15. Is there anything else you’d like to say about the triage system used here?

--------------------------------------------------------------------------------------------

Would you like to see a summary of what comes out of the interviews with staff?

Thank you for your time.
