# Supplementary material for: Process evaluation for complex interventions in health services research: analysing context, text trajectories and disruptions
Source: BMC Health Serv Res. 2016 Aug 19;16:407. doi: 10.1186/s12913-016-1651-8 (PMC4990981; doi:10.1186/s12913-016-1651-8)
Supplement: Additional file 2: — Patient interview guide. Description of data: Topic guide for interviews with patients participating in ESTEEM process evaluation (DOC 295 kb) [file 12913_2016_1651_MOESM2_ESM.doc]

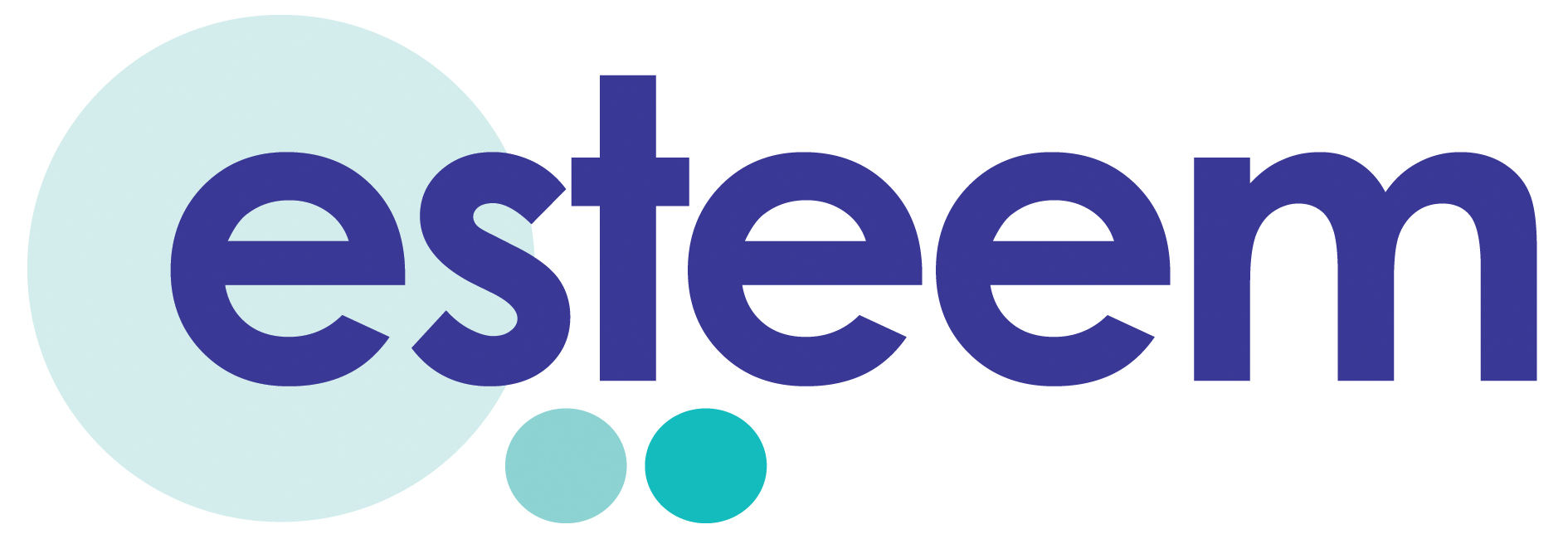
**Patient Interview Guide:**

## Preamble:

- Thank the patients for agreeing to meet/their time etc.
- Outline the (pilot) study aims.
- Check patients have read/understood the study information leaflet.
- Give them a chance to ask questions.
- Check that they wish to proceed with the interview.
- Complete/sign two consent forms (leave one with patients).
- Advise patients that we seek their experience/views, with no ‘right’ or ‘wrong’ answers.
- Advise them they can stop the interview at any point/decline to answer any questions.
- Advise them when audio-taping will begin.

## -------------------------------------------------------------------

## Interview questions:

You recently phoned your GP’s surgery to ask for a ‘same day’ appointment and were asked to take part in this study (check that patients agree with this).

1. Can you tell me what led you to contact your surgery on that particular occasion?

- prompt – appointment for self/child/other adult?

- was the appointment an emergency?

2. When you rang to ask for that appointment, what did you expect might happen?

3. To what extent were your expectations met?

- prompt - in what ways were your expectations met/unmet?

4. Did you end up coming into the surgery to see someone, after you rang, or was

your problem sorted out over the phone?

If you came into the surgery –

5. Who did you see?

- prompt – GP, nurse, other health professional?

6. How long did you have to wait to see that person? How satisfied were you with

this length of wait?

7. To what extent was your problem sorted out?

8. Do you feel you got to see the health professional that you wanted to?

9. How convenient did you find the same day appointment system overall?

10. How satisfied are you with the way you were treated from the point when you

first phoned the surgery onwards?

11. Is there anything else you’d like to say about your experience of getting a same day appointment?

- prompt – telephones;

reception staff;

clinical care;

convenience;

clinician/patient interaction;

overall ‘feel’ of the process

etc

----------------------------------------------------------------------------------------------

Would like to see a summary of what comes out of the conversations with patients?

Thank you for your time.
