# Supplementary material for: Process evaluation for complex interventions in health services research: analysing context, text trajectories and disruptions
Source: BMC Health Serv Res. 2016 Aug 19;16:407. doi: 10.1186/s12913-016-1651-8 (PMC4990981; doi:10.1186/s12913-016-1651-8)
Supplement: Additional file 3: Table S1. — Analysing disruptions in the implementation of primary care telephone triage. Description of data: Multi-modal transcript, including use of screenshot images (DOC 290 kb) [file 12913_2016_1651_MOESM3_ESM.doc]

**Analysing disruptions in the implementation of primary care telephone triage.**

The extract below is a multi-modal transcript taken from recordings obtained in one nurse-triage practice participating in ESTEEM.

| **Time**  **(Mins:Secs)** | **Nurse (N)/Patient (P1)** | **Talk** | **Screenshot of CDSS** |
| --- | --- | --- | --- |
| 00:11 | N: | How can I help you this morning | 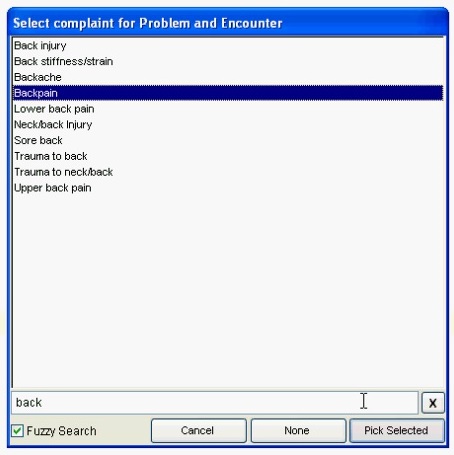  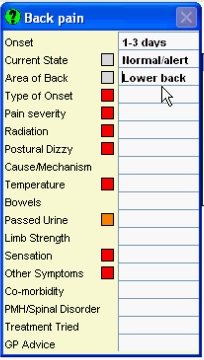  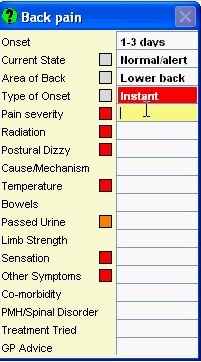 |
| 00:25 |  |  |
|  | P: | We:ll u:m I’ve (.) ↑on Fri:::day °huh° very ea::rly:: (.) I’ve had the most awful pai::ns (.) in my (.) lower (.) back (.) left hand si::de (.) now a few weeks ago:: I >had a< pai::n down my right side of my right le:g (.) from the knee downwards (.) .h so I don’t know whether it’s sciatica (.) but it’s very painfu::l |
| 00:36 | N: | .hh so it’s in you:r (.) ↑back, (.) lower back, (.) but going down your left leg. |
|  |  |  |
|  | **P:** | Um no the ↑right l- (0.4) ↑that’s on the left side yes but I had pains a few weeks ago on the right si::de |
| 00:49 | **N:** | So it’s what you’re phoning up today with thou:gh is (.) you’ve got pai::n down the left side of your back |
|  | **P:** | Ye::s |
| 00:56 | **N:** | °°Okay°° ummm |
|  |  | (.) |
|  | **P:** | °Yea::h° (0.4) I don’t whether you can give me any: (.) strong painkillers, or something, [cos it] |
| **01:02** | **N:** | [Well] we’d need to we’d need to revie:w you really so you’ve had it for about ↑three da::ys (.) ↑a:::nd (.) did it, (.) so it’s the lower l- (.) ↑↑do you feel it’s ↑around the ↑↑kidney a↓rea, |
|  | **P:** | Well no::: i- it’s u:::m |
| **01:16** | **N:** | It’s just the lower back |
|  | **P:** | It’s ↑ha::lf way down the back buttock I suppose it i:s |
| **01:21** | **N:** | ↑↑Oka::y, and i- it was a gradual on↑set or did it just suddenly sta::rt |
|  |  |  |
| **01:25** | **P:** | No i- it suddenly sta::rted and, (.) as I say I had to sort of actually shuffle alo::ng I couldn’t, |
| **01:33** | **N:** | Oh dear | 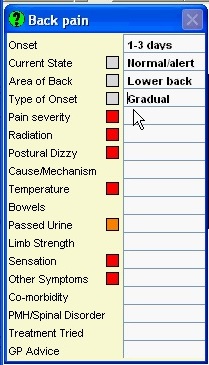 |
|  | **P:** | Put my foot (on it) or anythi::ng (.) fairly fa::r, |  |
| **01:36** | **N:** | So the ↑pain at the moment is nought is no pain ten is the worst ever where would you say your pain score would be |  |
|  |  | (.) |  |
| **01:43** | **P:** | My pai::::n? |  |
|  | **N:** | At the momen**t** | **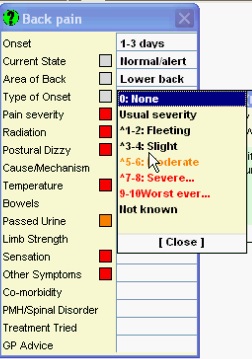** |
| **01:46** | **P:** | Well it’s still the:::re but I (.) I I’ve taken some paracetamol, |  |
|  | **N:** | O:::kay |  |
|  | **P:** | So it’s slightly better of course |  |
| **01:54** | **N:** | And you don’t feel that it’s moving down into the buttocks |  |
|  |  | (0.6) |  |
| **01:58** | **P:** | We::ll [I’d say it] (0.6) well I don’t kno::::w it = |  |
|  | **N:** | [(?) ] |  |
|  | **P:** | =(.) it’s as I sa:y it ha- halfway do::wn the buttock an- (.) not so far in I would think I don’t ↑know |  |
| **02:08** | **N:** | Okay that’s fi::ne | **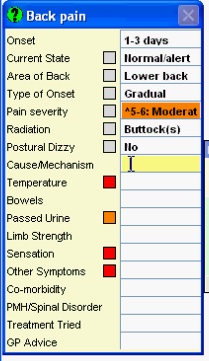** |
